# Investigation of the first carbon monoxide poisoning cluster associated with a hotpot restaurant in Thailand, 2023

**DOI:** 10.5365/wpsar.2026.17.1.1110

**Published:** 2026-03-25

**Authors:** Siriyakorn Thanasitthichai, Oranut Srihadom, Tanaporn Thongsim, Pasika Nonluecha, Kriangkrai Kampaiboon, Chuthamat Bodnok, Pawinee Doungngern

**Affiliations:** a Field Epidemiology Training Program, Ministry of Public Health, Nonthaburi, Thailand.; b Division of Occupational and Environmental Diseases, Department of Disease Control, Ministry of Public Health, Nonthaburi, Thailand.; c Pathum Thani Hospital, Pathum Thani, Thailand.; d Pathum Thani Provincial Public Health Office, Ministry of Public Health, Pathum Thani, Thailand.; e Division of Epidemiology, Department of Disease Control, Ministry of Public Health, Nonthaburi, Thailand.

## Abstract

**Objective:**

On 27 June 2023, the Thailand Department of Disease Control was notified of an incident of carbon monoxide poisoning related to a Thai-style hotpot restaurant. An outbreak investigation was performed to describe the incident, confirm its cause and sources of exposure, and provide preventive measures.

**Methods:**

The restaurant owner, restaurant guests and waiting staff were interviewed, and the medical records of hospitalized cases were reviewed. In an environmental survey, air quality parameters were measured, including temperature, relative humidity, carbon dioxide and carbon monoxide. Additionally, a simulation of the incident was conducted, and data were reviewed from previous poisoning incidents in Thailand.

**Results:**

There were 11 cases, all of whom were guests who dined in the same private dining room. The median age of cases was 28 years (range 2–62 years). Three cases were hospitalized and received hyperbaric oxygen therapy. The air changes in the dining rooms were below the recommended level. The incomplete combustion of charcoal in a poorly ventilated room led to carbon monoxide build-up, which caused the incident. The simulation experiment showed a high concentration of carbon monoxide (mean 183.16 ± 55.15 ppm), above the standard level. Ten similar poisoning incidents occurred between 2019 and June 2023, totalling 23 cases and 2 deaths; none occurred in a restaurant.

**Discussion:**

Charcoal use in poorly ventilated areas poses a health risk, especially for children. The use of charcoal stoves for hotpot cooking indoors is prohibited. Public health policy should mandate regular restaurant inspections to ensure compliance with occupational and environmental health standards.

Carbon monoxide (CO) is an odourless, colourless, non-irritant gas produced from an incomplete combustion of hydrocarbon fuels and substances. When inhaled, CO becomes toxic by binding and altering the functions of heme proteins in the haemoglobin, myoglobin and other components of oxygen-carrying pathways. The formation of carboxyhaemoglobin (COHb) severely impairs the oxygen-carrying capacity of blood and reduces oxygen release to the tissues. The primary targets of CO-induced injuries are the brain and heart, the organs with the highest oxygen demand. (*1*) Symptoms of CO toxicity include headache, dizziness, confusion, nausea, blurred vision, rhabdomyolysis, arrhythmias, myocardial infarction, loss of consciousness, seizure and coma. (*2*) Delivery of 100% oxygen is the mainstay treatment for CO poisoning. In addition, hyperbaric oxygen therapy should be considered in patients at high risk of severe symptoms to improve long-term neurological outcomes and survival. (*3*) Children, older adults, pregnant women and people with anaemia are more susceptible to CO toxicity, as are those with chronic respiratory, cerebrovascular or coronary heart diseases. (*4*)

Unintentional, non-fire related (UNFR) CO poisoning is a public health concern. While highly preventable, it is the leading cause of poisoning morbidity and mortality in many countries. (*5*) In 2007, an estimated 21 000 emergency department visits and 2300 hospitalizations in United States of America were attributed to UNFR CO poisoning. (*6*) They mainly occur in domestic settings and are more common during the autumn and winter months. (*7*, *8*) Fuel-burning appliances such as heating systems are responsible for the majority of incidents. (*9*-*11*) However, no previous studies have investigated the incidence or characteristics of CO poisoning in Thailand.

On 27 June 2023, the Division of Occupational and Environmental Diseases, Department of Disease Control, Ministry of Public Health, was notified of an incident of CO poisoning among restaurant guests who had dined in the same dining room of a Thai-style hotpot restaurant on the previous day in a province in the central region of Thailand. The Department of Disease Control, the Office of Disease Prevention and Control Region 4 Saraburi, Pathum Thani Hospital, and the local health-care team jointly investigated the incident on 28 June and 4 July 2023. In this report, we describe the incident, confirm the cause and sources of exposure, and provide recommendations for CO poisoning prevention.

## Methods

For the epidemiological investigation, active case-finding was initiated, and those who were in the restaurant at the time of the event were interviewed, including the restaurant guests and waiting staff who were in the private dining room on the day of the event, as well as the restaurant owner. The medical records of those who visited hospitals were reviewed.

Data on patients’ demographic characteristics, medical history, time and manner of CO exposure, clinical symptoms, laboratory results and treatments received were collected using a semi-structured questionnaire. Case definitions were adapted from the 2019 case definition of CO poisoning by the United States Centers for Disease Control and Prevention. (*12*) Suspected cases were defined as restaurant guests or staff who had been in the dining room on 26 June 2023 and who had at least one of the following symptoms: fatigue, nausea, vomiting, chest pain, difficulty breathing, blurred vision, headache, dizziness, confusion, light-headedness, loss of consciousness and a history of CO exposure. Confirmed cases were suspected cases who had a COHb level over 6% for non-smokers or over 12% for smokers.

A walk-through survey of the restaurant facilities was conducted. The air quality of the indoor and outdoor dining areas was monitored. The targeted parameters were temperature, relative humidity, carbon dioxide (CO_2_) and CO. Each sampling point was placed 1.1 m above the floor and away from potential sources of air pollution. The ventilation rate of dining rooms was determined by the number of air changes per hour, which equals the volumetric air flow rate divided by the volume of space. (*13*)

A 60-minute simulation was conducted using data from the cases to further analyse the air quality of the dining room at the time of the incident. Details on the types and number of possible sources of pollutants and the environmental conditions during which the CO poisoning occurred were obtained through interviews. Air temperature, relative humidity, CO_2_ and CO were measured using the Q-Trak Indoor Air Quality Monitor Model 7575 and a model IAQ Probe 982 (TSI Inc., Shoreview, MN, USA). For air velocity measurement, the Testo 400 – Universal IAQ Instrument and a 100-mm vane probe head (Testo SE & Co. KGaA, Titisee-Neustadt, Germany) were used. Devices were calibrated, and pre- and post-zero checks were done. Air quality data were recorded at each sampling point at an interval of 10 seconds for at least 15 minutes. The data were calculated as mean and standard deviation.

We also reviewed UNFR CO poisoning incidents recorded in the event-based surveillance system of the Thai Department of Disease Control from January 2019 to June 2023 to determine the number and characteristics of UNFR CO poisoning incidents in Thailand, using the same case definitions as in our epidemiological investigation.

## Results

### The restaurant

The hotpot restaurant has an outdoor dining area and a dining room (Dining Room A), as well as two private rooms (Private Rooms B and C). The CO poisoning incident took place in Private Room B. The restaurant had been open for 10 months at the time and serves a variety of Thai-Esan cuisine, including Thai-style hotpots cooked on burning charcoal stoves. Typically, no more than three hotpots are served in a private dining room at a time.

### Description of the incident

The restaurant did not have a record of guests’ names or contact numbers; therefore, we were only able to interview the affected guests and the waiting staff. There were 11 restaurant guests and four waiting staff in Private Room B on 26 June 2023. All restaurant guests were family members. Eleven restaurant guests met our case definitions (8 suspected and 3 confirmed cases), whereas none of the waiting staff were symptomatic. The median age of cases was 28 years (range 2–62 years). Three cases had underlying diseases (chronic hypertension, diabetes mellitus, asthma). None were pregnant. Demographic, treatment and outcome data of cases are shown in **Table 1**. The median duration of CO exposure was 140 minutes (range 90–140 minutes) among restaurant guests and 18 minutes (range 10–70 minutes) among waiting staff. The median time to symptom onset was 90 minutes (range 45–300 minutes) after first exposure. Dizziness (82%), headache (46%) and light-headedness (46%) were the most common symptoms.

**Table 1:** Demographic, treatment and outcome data of carbon monoxide poisoning cases in Private Room B of a hotpot restaurant, central Thailand, 2023

ID	Sex	Age (years)	Underlying diseases	Case status	Visit type	COHb level (%)	Treatment	Outcome at 1-week follow-up
**1**	**Female**	**58**	**T2DM, HT, DLP**	**Confirmed**	**IPD**	**33**	**HBOT**	**Mild headache, middle ear barotrauma grade II**
**2**	**Female**	**19**	**None**	**Confirmed**	**IPD**	**25**	**HBOT**	**Middle ear barotrauma grade II**
**3**	**Male**	**11**	**None**	**Confirmed**	**IPD**	**31**	**HBOT**	**Fully recovered**
**4**	**Female**	**40**	**None**	**Suspected**	**OPD**	**–**	**Supportive**	**Fully recovered**
**5**	**Male**	**62**	**T2DM, HT**	**Suspected**	**No**	**–**	**None**	**Mild fatigue**
**6**	**Female**	**54**	**None**	**Suspected**	**No**	**–**	**None**	**Fully recovered**
**7**	**Male**	**34**	**None**	**Suspected**	**No**	**–**	**None**	**Fully recovered**
**8**	**Female**	**28**	**None**	**Suspected**	**No**	**–**	**None**	**Fully recovered**
**9**	**Male**	**19**	**None**	**Suspected**	**No**	**–**	**None**	**Fully recovered**
**10**	**Male**	**19**	**Asthma**	**Suspected**	**No**	**–**	**None**	**Fully recovered**
**11**	**Female**	**2**	**None**	**Suspected**	**No**	**–**	**None**	**Fully recovered**

COHb: carboxyhemoglobin; DLP: dyslipidaemia; HBOT: hyperbaric oxygen therapy; HT: chronic hypertension; IPD: inpatient department; OPD: outpatient department; T2DM: type II diabetes mellitus.

**Fig. 1:**
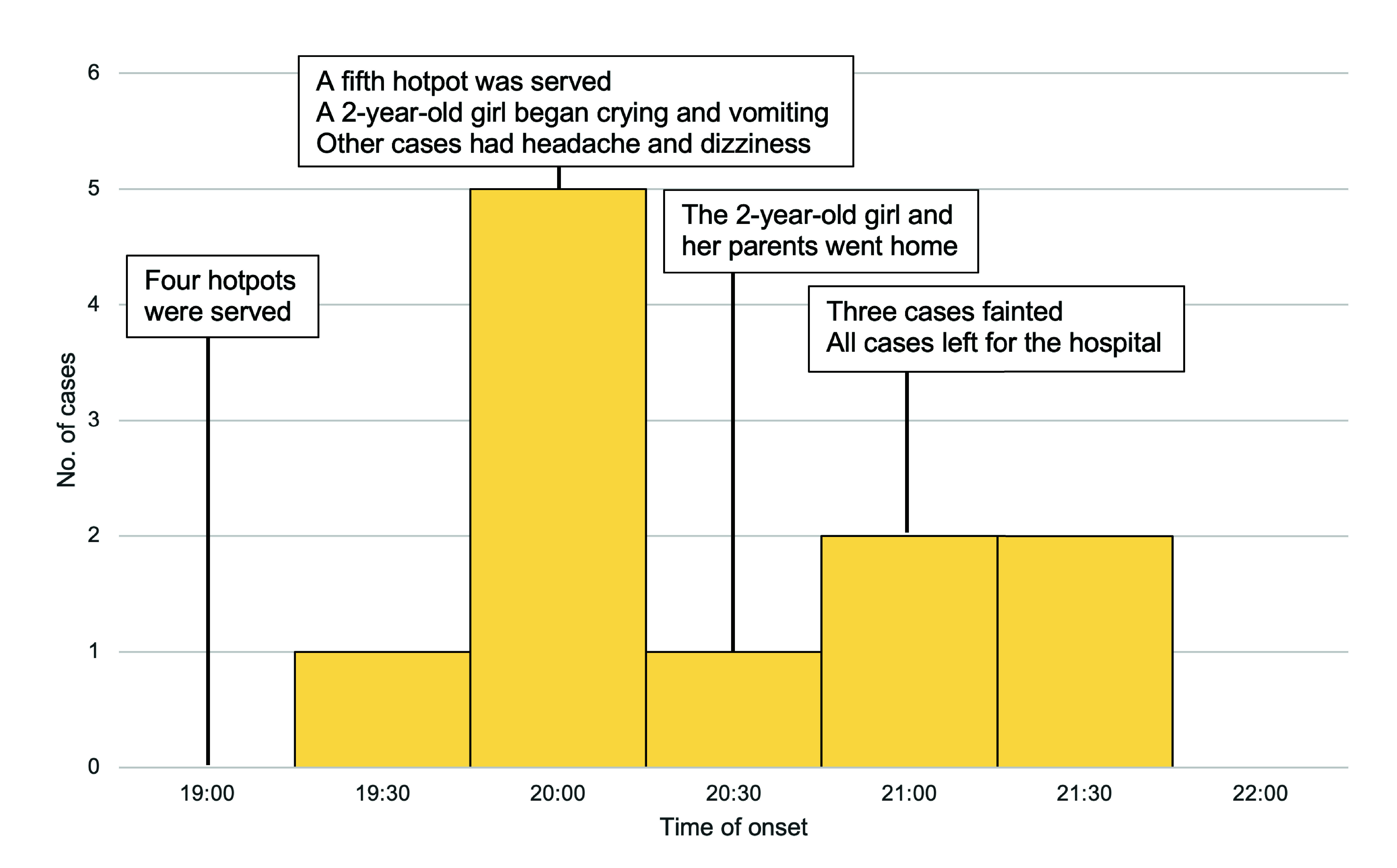
Number of carbon monoxide poisoning cases in a hotpot restaurant by time of onset, central Thailand, 26 June 2023 (*N* = 11)

The guests arrived at the restaurant around 19:00 and ordered five Thai-style hotpots. Four were served at the beginning and one was served an hour later. At 20:00, a 2-year-old girl started crying and vomiting, and her parents took her home at 20:30. Around that time, other guests in the room reported experiencing symptoms such as fatigue, headache and dizziness. At 21:00, three restaurant guests (11-year-old boy, 19-year-old woman, 58-year-old woman) fainted, so the family left for the hospital. No other guests entered Private Room B on that day (**Fig. 1**).

[**insert** Figure 1]

On arrival, the three patients with light-headedness were sent to the emergency department. Their COHb levels were 25%, 31% and 33%. They received hyperbaric oxygen therapy and were hospitalized for 24-hour observation. Two of them developed grade II middle ear barotrauma after treatment. Other cases had mild symptoms and were treated as outpatients. At 1-week follow-up, two cases had lingering headache and fatigue and nine had fully recovered (**Table 1**).

### Indoor air quality of the dining areas

The restaurant is in an urban area with moderate traffic intensity. No other sources of air pollution (for example, open chimneys) were observed nearby. All indoor dining rooms have mini-split air conditioning systems. There are 1–2 wall exhaust fans in each room.

During the environmental survey, the weather was cloudy with light rainfall. Daytime temperatures were up to 33 °C with a relative humidity of 55–89%. The air quality parameters of the dining rooms were measured while air conditioners and exhaust fans were on. The measurements were performed during the restaurant’s closing hours (13:00–16:00) at the request of the restaurant owner. The mean values of temperature, relative humidity, CO and CO_2_ of the dining rooms were within acceptable ranges according to the Notification of the Department of Health regarding Indoor Air Quality Monitoring Standards in Public Building B.E. 2565 (2022), except for the temperature of Private Room B, which was slightly higher than the recommendation. (*14*) However, the ventilation rates of the dining rooms were below the required 10 air changes per hour, ranging from 0.25 to 4.19 air changes per hour (**Table 2**).

**Table 2:** General information and air quality parameters of dining areas in the restaurant during the 60-minute simulation in Private Room B, central Thailand, 2023

Parameter	Thai IAQ standards (*14*)	Dining Room A	Private Room B	Private Room C	Outdoor zone
**Room volume (m^3^)**	**–**	**58.29**	**99.26**	**43.12**	**–**
**Capacity**	**–**	**12–18 guests**	**18–28 guests**	**6–12 guests**	**150–200 guests**
**Ventilation system**	**–**	**1 exhaust fan, sized 0.10 × 0.10 m**	**2 wall exhaust fans, sized 0.22 × 0.22 m**	**1 wall exhaust fan, sized 0.22 × 0.22 m**	**–**
**Temperature (°C)**	**24–26**	**25.90 ± 0.41**	**26.38 ± 0.62**	**24.55 ± 0.33**	**28.96 ± 0.58**
**Relative humidity (%)**	**50–65**	**63.53 ± 3.63**	**57.93 ± 1.16**	**63.25 ± 0.72**	**80.25 ± 6.19**
**CO (ppm)**	** ≤ 9**	**3.78 ± 0.22**	**0.08 ± 0.09**	**0.00 ± 0.00**	**0.01 ± 0.04**
**CO_2_ (ppm)**	** ≤ 1 000**	**544.00 ± 6.12**	**500.94 ± 47.16**	**406.04 ± 14.15**	**361.98 ± 12.38**
**Air changes per hour**	** > 10**	**0.25**	**3.15**	**4.19**	**–**

°C: centigrade; CO: carbon monoxide; CO_2_: carbon dioxide; IAQ: indoor air quality; ppm: parts per million.

### Simulation of CO poisoning incident

**Fig. 2:**
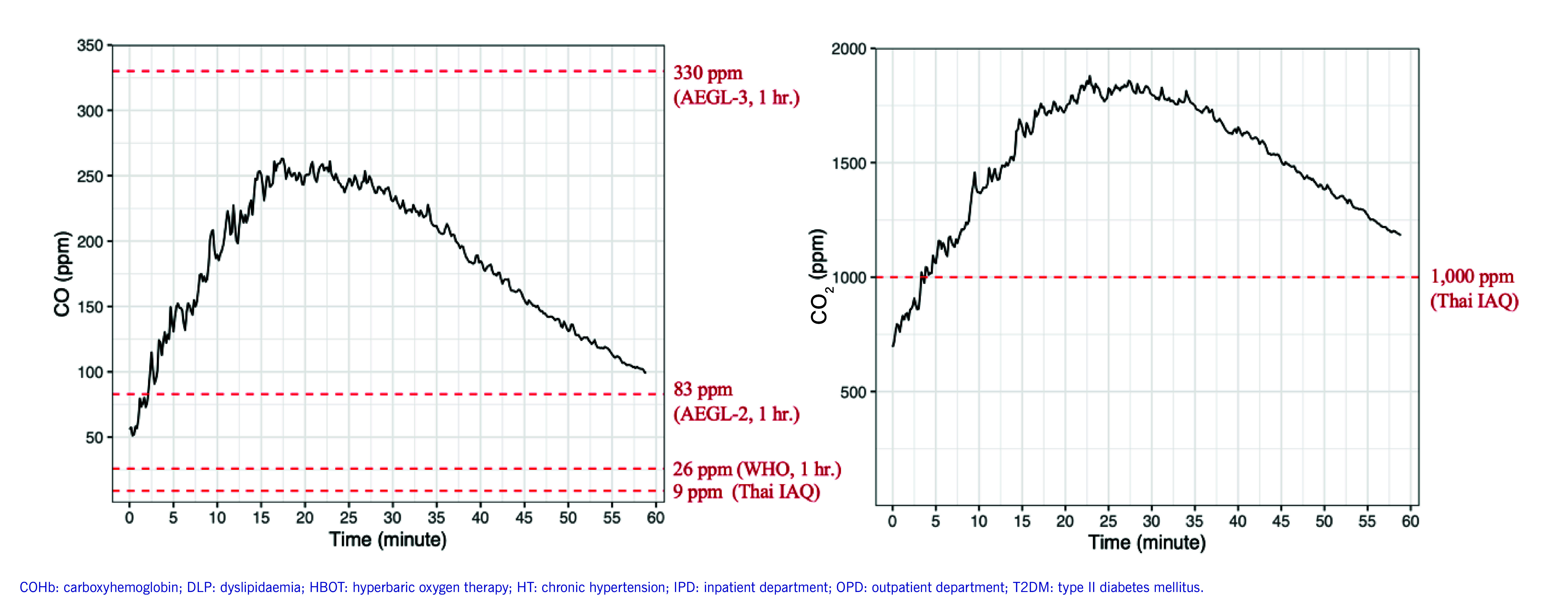
Carbon monoxide and carbon dioxide concentrations during the 60-minute simulation in Private Room B, central Thailand, 2023

A 60-minute simulation of the CO poisoning incident was conducted in Private Room B. Four charcoal stoves were lit, the air conditioner and wall exhaust fans were operating, and the door was closed. During the simulation, the mean CO was 183.16 ± 55.15 ppm (ppm) and the mean CO_2_ was 1502.54 ± 283.61 ppm (**Fig. 2**). The concentrations of CO and CO_2_ in the air were both above the acceptable limits set by the Thai Department of Health for indoor air quality. (*14*) Moreover, the CO concentrations exceeded the World Health Organization air quality guideline value (< 26 ppm in 1 hour) and the Acute Exposure Guideline Level 2 (AEGL-2) value (< 83 ppm in 1 hour) for CO. (*15*, *16*)

[**insert** Figure 2]

### Public health response

The restaurant owner was notified of the results of the analysis of the incident and advised that the use of indoor charcoal stoves was strictly prohibited. A visible sign has been posted at the restaurant's entrance to inform patrons of this policy. The floor plans of the restaurant were reviewed by municipal officials, and the restaurant was temporarily closed to undergo renovations to ensure compliance with environmental standards. A food hygiene and safety training course for local restaurant owners and staff was conducted, during which a module on CO poisoning was presented. An online public education campaign was launched to raise awareness of the risks of unintentional CO poisoning.

### Unintentional, non-fire related carbon monoxide poisoning in Thailand

Between 1 January 2019 and 30 June 2023, the Department of Disease Control's surveillance system showed 10 incidents of UNFR CO poisoning in Thailand, resulting in 23 cases and 2 deaths. The median number of cases per event was 1.5 (range 1–9). Nine incidents occurred in northern Thailand, and eight were caused by using gas water heaters in unventilated bathrooms in local resorts. Eight of the incidents occurred during the winter months. Notably, one incident involved the use of charcoal stoves for hotpot cooking in a camping tent. No prior reports of CO poisoning in restaurants had been documented.

## Discussion

We confirmed the first incident of CO poisoning in a food establishment in Thailand, which occurred at a Thai-style hotpot restaurant. The source of CO exposure was from charcoal stoves in which hotpots were cooked. Hotpot restaurants have been linked to multiple incidents of unintentional CO poisoning. Gaüzère et al. reported the mass CO intoxication of more than 50 people who dined in an unventilated Chinese hotpot restaurant. (*17*) Chua et al. reported another mass CO poisoning of 30 hotpot restaurant staff due to an exhaust fan malfunction. (*18*) Environmental studies in China found that nearly half of hotpot and barbeque restaurants studied had CO levels above the recommended indoor air quality standard. The CO levels were highest in hotpot restaurants that used charcoal as the main fuel. (*19*, *20*)

In the current incident, two children were the first to exhibit symptoms of CO poisoning. Children are more vulnerable to CO toxicity and experience symptoms much earlier than adults due to their elevated respiratory and metabolic rates, increased oxygen requirements and immature central nervous systems. (*21*, *22*) In a report by Klasner et al., children started having headache, dizziness and nausea at a COHb level as low as 7%. (*23*) In contrast, healthy adults would only feel occasional temporal headache at a COHb level of around 20%. (*24*) Additionally, children are prone to the neurotoxic effects of CO. According to studies in China, Taiwan (China) and the United States, approximately 60–80% of children who presented to the hospital with CO poisoning exhibited acute neurological manifestations, with symptoms ranging from varying degrees of consciousness changes and tremor to ataxia and seizures. (*25*-*27*)

From the environmental survey, we found that the air change rates of dining rooms were significantly below the standards set by the Department of Health. (*14*) Poor air changes together with the use of multiple charcoal stoves led to rapid accumulation of CO and other pollutants. During the simulation, the CO concentrations exceeded the AEGL-2 value in less than 5 minutes. CO exposure at this level could result in serious health damage, particularly in susceptible populations. For example, it could cause significant cardiac changes and reduce the time to onset of angina in patients with coronary artery disease. (*28*, *29*) As for CO_2_, short-term exposure to concentrations above 1000 ppm is not immediately dangerous, but it can lead to a reduction in cognitive performance. (*30*) Our findings highlight the dangers of using charcoal in poorly ventilated areas.

The findings of this study should be interpreted with caution due to some limitations. First, for safety reasons, we were unable to simulate the exact event with people in the private room. Second, the environmental survey was conducted during the restaurant’s closing hours. Therefore, the air pollution levels that were detected may have been lower than those that would have been present during the restaurant's operating hours, when cooking activities were taking place. Third, the lack of laboratory results for patients with mild symptoms limited our ability to confirm the diagnosis of CO poisoning.

Future policy should mandate routine inspections of all restaurants to ensure compliance with occupational and environmental health standards. These inspections should verify adequate ventilation of indoor dining areas and proper venting of fuel-burning appliances. Restaurants should also be required to train their staff on the hazards of CO poisoning and how to prevent it. By implementing these measures, the risk of unintentional CO poisoning in restaurants could be effectively reduced.

We reported a CO poisoning incident associated with a hotpot restaurant on 26 June 2023. All 11 cases were restaurant guests, aged 2–62 years. Three cases were hospitalized and received hyperbaric oxygen therapy. No deaths were reported. Charcoal stoves were the source of CO exposure. From an environmental survey, we found that the restaurant’s indoor dining areas had very low air exchange rates, which contributed to the accumulation of CO. The use of indoor charcoal stoves was prohibited. The restaurant floor plan and ventilation system were reviewed by local authorities and renovated. In addition, an online public awareness campaign on unintentional CO poisoning was launched. The routine inspection of restaurants and the provision of training sessions on CO poisoning are recommended as future policy interventions to mitigate the risk of CO poisoning in restaurants.
